# Dropout From Trauma-Focused Treatment for PTSD in a Naturalistic Setting

**DOI:** 10.32872/cpe.14491

**Published:** 2025-02-28

**Authors:** Verena Semmlinger, Keisuke Takano, Larissa Wolkenstein, Antje Krüger-Gottschalk, Sascha Kuck, Anne Dyer, Andre Pittig, Georg W. Alpers, Thomas Ehring

**Affiliations:** 1 Department of Psychology, LMU Munich, Munich, Germany; 2 Human Informatics and Interaction Research Institute (HIIRI), National Institute of Advanced Industrial Science and Technology (AIST), Tsukuba, Japan; 3 Institute of Psychology, WWU Münster, Münster, Germany; 4 Central Institute of Mental Health, ZISG Mannheim, Mannheim, Germany; 5 Translational Psychotherapy, Institute of Psychology, Georg-August-University of Göttingen, Göttingen, Germany; 6 Department of Psychology, School of Social Science, University of Mannheim, Mannheim, Germany; 7 German Center for Mental Health (DZPG), Munich, Germany; Philipps-University of Marburg, Marburg, Germany

**Keywords:** treatment dropouts, posttraumatic stress disorder, prediction, psychotherapy, clinical practice, naturalistic setting

## Abstract

**Background:**

Although evidence-based interventions for posttraumatic stress disorder (PTSD) are highly effective, on average about 20% of patients drop out of treatment. Despite considerable research investigating PTSD treatment dropout in randomized controlled trials (RCTs), findings in naturalistic settings remain sparse.

**Objective:**

Therefore, the present study investigated the frequency and predictors of dropout in trauma-focused interventions for PTSD in routine clinical care.

**Method:**

The sample included *n* = 195 adults with diagnosed PTSD, receiving trauma-focused, cognitive behavioral therapy in routine clinical care in three outpatient centers. We conducted a multiple logistic regression analysis with the following candidate predictors of dropout: patient variables (e.g., basic sociodemographic status and specific clinical variables) as well as therapist’s experience level and gender match between therapist and patient.

**Results:**

Results showed a dropout rate of 15.38%. Age (higher dropout probability in younger patients) and living situation (living with parents predicted lower dropout probability compared to living alone) were significant predictors of dropout. Dropout was not significantly associated with the therapist’s experience level and gender match.

**Conclusions:**

In conclusion, routinely assessed baseline patient variables are associated with dropout. Ultimately, this may help to identify patients who need additional attention to keep them in therapy.

## Highlights

- About 15% of patients receiving PTSD treatment in routine clinical care dropped out.
- This rate is lower than found in previous studies.
- Age and living situation were the only variables related to dropout.

Evidence-based interventions for posttraumatic stress disorder (PTSD) have been shown to be highly effective (e.g., Mavranezouli et al., 2020). However, about 20% of patients receiving an intervention for PTSD drop out of treatment (e.g., Varker et al., 2021). As treatment dropout can lead to lower treatment effectiveness and reduced probability of improvement (Barrett et al., 2008; Varker et al., 2021), PTSD treatment dropout is an important clinical challenge. On a general level, dropout can be defined as termination of an initiated treatment before the symptoms that had caused the patient to seek treatment have been alleviated (Swift et al., 2009; Swift & Greenberg, 2012). Despite repeated efforts to establish a common standard, there remains a lack of consensus in the literature regarding the operationalization of dropout, resulting in different variants being observed (e.g., Barrett et al., 2008; Imel et al., 2013). One criterion that is common in many different operationalization methods is that dropout is a unilateral decision by the patient without mutual agreement or discussion of the decision with the therapist (Swift et al., 2012). In clinical practice, therapist judgement has been discussed for many years as a preferred operationalization method (Swift & Greenberg, 2012; Wierzbicki & Pekarik, 1993) that can be combined with an objective measure to ensure reliability and comparability (Semmlinger & Ehring, 2022).

Previous research has focused on estimating the prevalence of dropout from psychological treatment in randomized controlled trials (RCTs). Across different mental disorders, a large-scale meta-analysis found a weighted average dropout-rate of 19.7%, 95% CI [18.7, 20.7] (Swift & Greenberg, 2012). The average dropout rate reported from evidence-based treatments for PTSD is comparable to this general dropout rate. In a recent meta-analysis investigating dropout from guideline-recommended psychological treatments for PTSD in RCTs, Varker et al. (2021) reported an average dropout rate of 20.9%, 95% CI [17.2, 24.9]. Similar dropout rates have been estimated by other previous meta-analyses that focus on a wider range of treatment orientations and settings (e.g., Imel et al., 2013: 18.3%, 95% CI [14.8, 21.8]; Lewis et al., 2020: 16%, 95% CI [14, 18]). While there is a vast body of research investigating dropout in RCTs, less is known about dropout rates from treatment for PTSD in routine clinical care. In a systematic review investigating dropout from outpatient treatment for PTSD in a sample of veterans with combat-related PTSD, Goetter et al. (2015) estimated a dropout rate of 36%, 95% CI [26.2, 43.9]. A recent meta-analysis including both RCTs and non-RCTs reported a weighted average dropout rate of 41.5% from trauma-focused CBT for PTSD (Mitchell et al., 2022). It is worth noting that, due to the focus of their analysis, Mitchell et al. (2022) only reported the average dropout rate across all studies and did not include information on the weighted dropout rates for RCTs and non-RCTs separately. Dropout rates for the included non-RCT studies were 35%, 67.5%, and 72.2% (Mitchell et al., 2022).

For dropout from PTSD treatment a number of predictors have been discussed. First, baseline PTSD symptom severity might influence dropout, evidence however is mixed. While Varker et al. (2021) did not find a significant effect, Mitchell et al. (2022) showed higher clinician-rated baseline PTSD symptom severity scores in patients dropping out of treatment compared to completers (Hedge’s *g* = .50, 95% CI [-.95, -.04], *p* < .05). It is worth noting that this effect applied only to clinician-rated but not to self-rated PTSD severity. Zandberg et al. (2016) added to these findings by examining the influence of the rate of improvement on dropout as a function of symptom severity. The authors showed that for patients with high baseline severity, high dropout rates were associated with both very fast and very slow PTSD improvement, in contrast to patients with low baseline severity, who showed high dropout rates only with fast improvement. The loss of motivation and reduction in the credibility of treatment caused by slow improvement of PTSD symptoms might result in a higher risk of dropout in patients with high PTSD severity (Zandberg et al., 2016).

Second, comorbidity is often discussed as a possible predictor, especially comorbid depression, generalized anxiety disorder (GAD), alcohol disorder, and borderline personality disorder (BPD) (e.g. Steindl et al., 2003). However, the findings are contradictory and potential mechanisms are still unknown (e.g., Angelakis & Nixon, 2015; Mitchell et al., 2022; Snoek et al., 2021). As possible explanations, different studies have discussed depressed patients’ reduced ability for emotional processing (Angelakis & Nixon, 2015) or the possible exacerbation of PTSD symptomatology and the increase of psychosocial impairment as a result of comorbid BPD (Frías & Palma, 2015). Specifically, with regard to dropout, a handful of studies have reported an effect of co-occurring depression (e.g., Zayfert et al., 2005), anxiety (e.g., McDonagh et al., 2005; van Minnen et al., 2002), or comorbid personality disorder (e.g., McDonagh et al., 2005) on dropout. However, recent large-scale meta-analyses did not find a significant relationship between comorbidity and dropout from PTSD treatment (Mitchell et al., 2022; Snoek et al., 2021; Varker et al., 2021).

Third, other pretreatment clinical variables might be associated with dropout in PTSD treatments. However, results to date are inconsistent and findings only rely on few studies. Possible predictors are difficulties in emotional regulation (no effect: Belleau et al., 2017; Shnaider et al., 2022; effect: Bremer-Hoeve et al., 2023; Gilmore et al., 2020), anger (no effect: Hinton et al., 2022; van Minnen et al., 2002; mixed results: Rizvi et al., 2009), impaired social functioning (effect: Zayfert et al., 2005), dissociative symptoms (no effect: Hagenaars et al., 2010), and childhood trauma (effect: Miles & Thompson, 2016; mixed results: Resick et al., 2014; no effect: van Minnen et al., 2002). In addition, the patient’s trauma response and maladaptive processing (e.g. avoidance, rumination, overgeneralization) may be associated with dropout (Alpert et al., 2020; Shayani et al., 2023). Alpert et al. (2020) found that more negative emotions and ruminative processing predicted lower dropout, whereas overgeneralization was associated to higher dropout. In contrast, Shayani et al. (2023) did not find an effect of overgeneralization, ruminative processing, and negative emotions, but did find that higher levels of avoidance were associated with higher dropout.

Concerning sociodemographic variables, only for the variable age is there a reasonable indication that younger age might be predictive for dropout in PTSD treatment (Garcia et al., 2011; Goetter et al., 2015; Rizvi et al., 2009). However, in two recent meta-analyses, none of the sociodemographic variables (including age) was found to be a consistent predictor across studies (Lewis et al., 2020; Varker et al., 2021).

The majority of studies investigating dropout in PTSD treatment have used an RCT design. Therefore, much less is known about dropout in naturalistic settings. To our knowledge, there is only one review with a veterans sample (Goetter et al., 2015) and few studies (Garcia et al., 2011; van Minnen et al., 2002) specifically investigating dropout in routine clinical care. Transferring results from efficacy studies (RCTs) to naturalistic therapeutic settings might be problematic (Leichsenring, 2004; Schindler et al., 2011). Despite the well-known strength of RCTs it has been discussed whether randomization in RCTs and the strict use of diagnosis specific treatment manuals impose artificial conditions that do not reflect the complexities of clinical practice. Therefore, naturalistic studies are required (Leichsenring, 2004).

The aim of the present study was to investigate the frequency and predictors of dropout in trauma-focused, guideline-recommended interventions for PTSD in routine clinical care. Due to the lack of research on the prevalence and predictors from PTSD treatment in naturalistic settings, our analyses followed an exploratory approach.

## Method

### Participants

Data was assessed at three university-based outpatient centers providing treatment for PTSD in Germany, located at LMU Munich (Dataset 1) as well as the University of Münster and the Otto Selz Institute at the University of Mannheim (Dataset 2). The sample consisted of 195 adult patients receiving treatment for PTSD. All data was collected as part of effectiveness studies evaluating trauma-focused cognitive behavioral therapy (TF-CBT) for PTSD in routine clinical care (previous, different analysis only on Dataset 2: Krüger-Gottschalk et al., 2024; Schumm et al., 2022, 2023). At pretreatment, all patients met DSM-5 diagnostic criteria for PTSD assessed via the Clinician-Administered PTSD Scale for DSM-5 (CAPS-5) (Weathers, Blake, et al., 2013), and were between 18 and 65 years old. Only participants who had already terminated their treatment at the respective institution and had attended at least one treatment session were included in the study. Exclusion criteria included current psychotic disorder, current substance dependence, or current suicidal intent (First, Williams, Karg, & Spitzer, 2016). Sociodemographic and clinical characteristics of the sample are presented in Table 1.

### Treatment

Treatment in all outpatient centers consisted of trauma-focused cognitive behavioral therapy following the same treatment manual. Due to the naturalistic setting of the study, no randomization took place and there was no control condition. The treatment manual is based on empirically tested therapy concepts (especially Ehlers & Clark’s cognitive therapy approach, Ehlers & Wild, 2022, as well as DBT-PTSD principles, Bohus et al., 2020) and follows a modularized phase-based approach (see also Ehring, 2019). It includes three consecutive phases. Phase 1 can be summarized as preparation for trauma-focused therapy, including providing a theoretical rationale, increasing treatment motivation, or reducing risky or self-destructive behavior where needed. Phase 2 consisted of the trauma-focused interventions. Therapists could choose between different trauma-focused interventions, including Prolonged Exposure, cognitive therapy, Imagery Rescripting, trigger analyses and discrimination training, as well as cognitive interventions targeting dysfunctional assumptions. Phase 3 was the final phase of treatment and focused on improving quality of life, resuming activities, and relapse prevention. The treatment plan was intended to take each patient through all three phases, with the number of sessions required for each phase and the selection of modules within each phase varying from patient to patient. Depending on the current symptomatology, deviations from this phase structure had to be made in individual cases.

Treatment sessions were usually provided on a weekly basis, with a regular session duration of 50 minutes. The overall average treatment length was *M* = 36.6 sessions (*SD* = 23.4). The average treatment length for dropout cases was *M* = 23.3 sessions (*SD* = 17.5) and *M* = 39.3 (*SD* = 23.5) for patient who did not drop out. On average patients underwent *M* = 5.0 (*SD* = 1.3) preparatory sessions. This is higher than typically reported in RCTs for PTSD, whereas 12 – 16 sessions are more frequently used. In the German healthcare system patients are permitted to receive up to 80 treatment sessions. Therefore, the reported number of sessions used in our study is typical of the German healthcare system. Second, PTSD treatment in RCTs is often provided in 90-100 min sessions, which means that the treatment dose received in the current study is not that different to typical RCT settings. The treatments were conducted by either licensed CBT therapists (39.2%) or psychotherapists in training (60.8%) employed at the outpatient centers. Supervision by a CBT therapist with expertise in PTSD treatment was regularly provided, on average at every second session. Given the naturalistic nature of the study, it was not feasible to implement formal fidelity checks. The majority of the therapists were female (86.4%).

### Measures

The baseline assessment included sociodemographic data, namely age, gender, marital status, living situation, and education. Clinical variables were assessed using clinical interviews and psychometric questionnaires. In addition, two therapist variables, i.e., experience level and gender match, were coded as potential predictors of dropout. For each patient, we revised the patient files, analyzing the therapeutic session protocols.

#### Dropout

Dropout was operationalized using the therapist’s judgement, and the termination had to be initiated by the patient, without a mutual agreement that termination was the best choice. Therapists routinely documented this information in patient files on a treatment termination form. In exceptional cases, where no information was provided, we used an elaborate file analysis, i.e., analyzing the three last session protocols for each respective patient, to retrieve the information needed. If no or only ambiguous information could be obtained, the patient was excluded from the study.

#### Clinician-Administered PTSD Scale for DSM-5 (CAPS-5)

The CAPS-5 (Weathers, Blake, et al., 2013; German translation by Schnyder, 2013) is a structured diagnostic interview that assesses posttraumatic stress symptoms in the past month. Symptoms are rated on a five-point Likert scale ranging from 0 = absent to 4 = extreme, with a rating of 2 or higher indicating the presence of a symptom (Weathers et al., 2018). The presence of at least one symptom per cluster “intrusive symptoms” and “avoidance”, and at least two symptoms per cluster “changes in mood and cognition” and “hyperarousal” indicated the presence of a PTSD diagnosis. The CAPS-5 is a gold-standard clinical interview with good reliability and validity (Weathers et al., 2018).

#### Structured Clinical Interview for DSM (SCID)

The SCID (First, Williams, Karg, & Spitzer, 2016; Wittchen et al., 1997) was used to assess the presence of comorbid disorders. The SCID for personality disorders (First, Williams, Smith Benjamin, & Spitzer, 2016; Fydrich et al., 1997) was administered to assess the presence of comorbid personality disorders. The SCID is a gold-standard clinical interview to assess diagnostic criteria according to the DSM. For each disorder, interview questions along the DSM criteria allow the rating of diagnostic symptoms as present or absent.

#### PTSD-Checklist for DSM-5 (PCL-5)

The PCL-5 (Weathers, Litz, et al., 2013; German version by Krüger-Gottschalk et al., 2017) was used to assess posttraumatic symptom severity. The assessment consists of 20 items, corresponding to the DSM-5 PTSD criteria. Distress caused by each symptom is rated on a five-point Likert scale ranging from 0 = not at all to 4 = extremely. Symptom severity was obtained as a sum score of all 20 items (range 0 to 80). The German PCL-5 has demonstrated high internal consistency (Cronbach’s α = .95) (Krüger-Gottschalk et al., 2017). In the current study, internal consistency was also high (α = .87). Please note that Cronbach’s alpha for all analyzed questionnaires was calculated on the non-imputed dataset.

#### Childhood Trauma Questionnaire (CTQ-28)

Exposure to traumatic childhood experiences was assessed with the CTQ-28 (Bernstein et al., 2003; German version by Klinitzke et al., 2012). The CTQ-28 is a self-report questionnaire consisting of 28 items, rated on a five-point Likert scale ranging from 1 = never true to 5 = very often true. A sum score for all items (range 25 to 128) was calculated. The German CTQ-28 shows overall good psychometric properties. The internal consistency for the four subscales without physical neglect is high (α ≥ .80), while the physical neglect subscale shows weak internal consistency (α = .55) (Klinitzke et al., 2012). In the current study, internal consistency was good (α = .95) for the total CTQ score.

#### Inventory of Interpersonal Problems (IIP-32)

The IIP-32 was used to assess interpersonal problems (Horowitz et al., 2000; German version by Thomas et al., 2011). The self-report questionnaire contains 32 items, assessing interpersonal behavior that the participant either finds difficult or shows in excess. The items are rated on a five-point Likert scale ranging from 0 = not at all to 4 = extremely. In the parent studies, different item versions of the questionnaire were used (IIP-127, IIP-64, IIP-32). For the main analyses, we used the IIP-32 version and narrowed the long versions down to the IIP-32. We calculated the IIP-32 total score as the mean of the eight scale scores (Horowitz et al., 2000). The internal consistency of the German IIP-32 was rated as satisfactory to good; for the individual scales it ranged from α = .60 to α = .83 (Thomas et al., 2011). In the current study the internal consistency for the total IIP-32 was high (α = .90).

#### Dissociative Experience Scale (DES)

Dissociative symptoms were assessed with the Dissociative Experience Scale (DES) (Bernstein & Putnam, 1986; German version by Spitzer et al., 2004, called FDS-20). The DES is a 20-item self-report questionnaire. Items are rated on a scale ranging from 0% (never) to 100% (all the time). We used the total mean score to determine the overall dissociation. The DES showed good psychometric measures and the internal consistency was α = .93 (Spitzer et al., 2004). In the current study internal consistency was high α = .93.

#### Posttraumatic Cognitions Inventory (PTCI) and Interpretation of Symptoms Inventory (IPSI)

Posttraumatic cognitions were assessed using a combined version of the PTCI (Foa et al., 1999) and the IPSI (Dunmore et al., 1999) (German versions by Ehlers & Boos, 2000). The self-report questionnaire assesses negative cognitions and beliefs in response to a traumatic experience (PTCI) and to posttraumatic symptoms (IPSI). The 48 items are rated on a seven-point Likert scale ranging from 1 = totally disagree to 7 = totally agree. We used the total sum score for PTCI and the IPSI mean (Ehlers, 1999). The German PTCI has demonstrated high internal consistency of α = .95 and good overall psychometric properties (Müller et al., 2010). The internal consistency reported for the IPSI was α = .84 (Dunmore et al., 2001). In the current study internal consistency was high for PTCI (α = .92) and IPSI (α = .92).

#### Difficulties in Emotion Regulation Scale (DERS)

Emotional dysregulation was assessed with the self-report questionnaire DERS (Gratz & Roemer, 2004; German version by Ehring et al., 2008). The 36 items are rated on a five-point Likert scale ranging from 1 = almost never to 5 = almost always. We used the DERS sum score (range 36 to 180) to determine possible difficulties in emotion regulation. The German version of the DERS has an excellent internal consistency of α = .96 (for the sum score) (Kruse et al., 2024). In the current study, internal consistency was excellent, α = .94.

### Procedure

The studies were approved by the local ethics committees at the LMU Munich, University of Münster, and the University of Mannheim. All three outpatient centers are specialized in the treatment of patients with trauma-related disorders. Participants referred to these centers were screened for eligibility. If eligible, participants received detailed information about the respective study, and written informed consent was obtained. Due to the naturalistic setting, participants were not randomized to different conditions but received standard care (see treatment). After the baseline assessment had taken place, the treatment was initiated at the next possible date.

All candidate predictor variables were assessed at baseline. The baseline assessment session consisted of clinical interviews (CAPS-5; SCID) as well as sociodemographic and clinical questionnaires. As treatment was delivered in a naturalistic setting, a substantial effort was made to prevent premature termination of treatment as part of the standard procedure. In the case of excused absence, a new appointment offer was made; in the case of unexcused absence, patients were called by the therapists to make a new appointment. If no contact could be made after several attempts, a letter was sent asking the patient to get in contact within a defined period of time to guarantee continued access to treatment. If the patient clearly expressed the desire to discontinue treatment, no further attempts to contact them were made.

### Statistical Analyses

All statistical analyses were conducted using R (Version 4.2.0). Datasets from two parent studies were merged for the current analyses. The dropout rate was calculated as the proportion of the patients who dropped out to the total number of patients who had started the treatment. There was a notable amount of missing data in some questionnaires (*M* = 7%, *SD* = 4%, max = 27%). The missing data was assumed to be missing at random (MAR) (Bhaskaran & Smeeth, 2014), and was imputed using the iterative procedure of conditional multiple imputation technique on an item level, i.e., before calculating the respective sum score. Conditional multiple imputation was realized by the five-step procedure proposed by Rubin (1976) and Kropko et al. (2014), using the R *Multivariate Imputation by Chained Equations (mice)* package (van Buuren & Groothuis-Oudshoorn, 2011). The number of multiple imputations as well as the number of iterations were set to five (m = 5, maxit = 5), and we used predictive mean matching (pmm) as the imputation method for continuous variables and the logistic regression (logreg) as the imputation method for dichotomous variables. We conducted a sensitivity analysis to ensure that the results were not affected by multicollinearity due to highly correlated items in the dataset or by the use of the multiple imputed dataset for our main analysis.

First, we tested the differences in demographics and baseline symptom levels between patients who dropped out and those who did not. Next, zero-order associations were examined between dropout and the predictors of interest using point-biserial correlation on the imputed data. We then conducted a multiple logistic regression analysis (maximum likelihood estimation; imputed data) to investigate the unique effects of the variables on dropout after controlling for the effect of the other variables in the model. The level of significance was set as α = .05. We included the following variables as potential predictors of dropout (all assessed at the beginning of treatment): age, gender, marital status, living situation, education, posttraumatic symptom severity (PCL), exposure to traumatic childhood experiences (CTQ), interpersonal problems (IIP), overall dissociation (DES), posttraumatic cognitions in response to the traumatic experience (PTCI) and to posttraumatic symptoms (IPSI), emotional dysregulation (DERS), number of previous treatments (outpatient and inpatient), number of comorbid disorders (all comorbid disorder), comorbid personality disorder, therapist’s experience level (registered vs. in training), and gender match.

Although our primary focus was on the effects of each predictor on dropout, we were interested in how well the logistic regression model would predict dropout. We evaluated the prediction performance using leave-one-out cross-validation on the imputed datasets. The following three performance measures were computed (as medians across imputed datasets): accuracy (i.e., the number of patients who were correctly identified by the model as dropouts or non-dropouts divided by the total number of patients), sensitivity (i.e., the number of dropouts correctly identified as dropouts by the model divided by the number of dropouts), and specificity (i.e., the number of non-dropouts correctly identified as non-dropouts divided by the number of non-dropouts). In addition, Receiver Operating Characteristic (ROC) analysis was performed to evaluate the discriminatory power of the logistic regression model. The area under the ROC curve (AUC) was calculated to summarize the overall performance of the model, again as median AUC across the multiple imputed datasets. The AUC typically ranges from 0 to 1, with 1 indicating the perfect separation and with 0.5 meaning random separation (or poor prediction performance).

## Results

### Descriptives and Demographics

The sample consisted of 195 patients, with a mean age of 36.14 years (*SD* = 13.02 years). The majority of patients were female (75.9%). Ninety-six patients (56.8%) had at least one comorbid disorder. The mean baseline PTSD symptom severity (PCL) was *M* = 46.2 (*SD* = 14.5), indicating a high severity of PTSD symptoms. Patients in the sample experienced a variation of traumatic events, including accidental trauma, victimization, or trauma predominantly related to death threat. There was a significant association between dropout and age (see Table 1), but not with respect to the other variables studied. The descriptive statistics for all demographic and clinical measures of the sample are presented in Table 1.

**Table 1:** Descriptive Statistics of the Sample, of Dropouts, and of No Dropout at Baseline

Variable	Total	Dropout	No Dropout	*t* or χ^2^ (*p*)
	*n* (%) / *M* (*SD*)	*n* (%) / *M* (*SD*)	*n* (%) / *M* (*SD*)	
Gender^a^				0.35 (.56)
Female	148 (75.9%)	21 (70.0%)	127 (77.0%)	
Male	47 (24.1%)	9 (30.0%)	38 (23.0%)	
**Age in years^b^**	36.1 (13.02)	29.97 (10.11)	37.28 (13.21)	**3.40 (.001)**
Marital status^c^				0.73 (.70)
Single	112 (59,6%)	19 (65.5%)	93 (58.5%)	
Married	58 (30.8%)	7 (24.1%)	51 (32.1%)	
Divorced/widowed	18 (9.6%)	3 (10.4%)	15 (9.4%)	
Living situation^b^				3.90 (.27)
Alone	41 (21.9%)	7 (24.1%)	34 (21.4%)	
With partner	106 (56.7%)	14 (48.3%)	92 (57.9%)	
With parents	23 (12.3%)	2 (10.3%)	21 (13.2%)	
Other	17 (9.1%)	5 (17.2%)	12 (7.5%)	
Highest education level^d^				4.15 (.25)
University degree	35 (18.5%)	3 (10.0%)	32 (20.1%)	
High school^†^	35 (18.5%)	9 (30.0%)	26 (16.4%)	
Secondary school^‡^	102 (54.0%)	16 (53.3%)	86 (54.1%)	
Other	17 (9.0%)	2 (6.7%)	15 (9.4%)	
Previous treatment^e^				0.63 (.43)
yes	106 (58.6%)	14 (50.0%)	92 (60.1%)	
no	75 (41.4%)	14 (50.0%)	61 (39.9%)	
Comorbid PD^f^				< .001 (1.0)
yes	15 (8.6%)	2 (6.9%)	13 (8.9%)	
no	160 (91.4%)	27 (93.1%)	133 (91.1%)	
**Number of CD^g^**	0.98 (1.1)	0.89 (0.91)	0.99 (1.13)	0.54 (.60)
Gender match^h^				0.02 (.89)
Match	107 (73.3%)	19 (70.4%)	88 (73.9%)	
No match	39 (26.7%)	8 (29.6%)	31 (26.1%)	
Approval therapist^i^				0.02 (.89)
Licensed	56 (39.2%)	11 (42.3%)	45 (38.5%)	
Non-licensed	87 (60.8%)	15 (57.7%)	72 (61.5%)	
Clinical measures^a^				
PCL-5	46.2 (14.5)	47.0 (12.2)	46.1 (15.1)	-0.33 (.74)
CTQ-28	55.2 (22.9)	49.6 (15.9)	56.2 (24.2)	1.46 (.15)
IIP-32	1.6 (0.6)	1.6 (0.5)	1.7 (0.7)	0.54 (.59)
DES	2.0 (1.8)	2.2 (1.5)	2.0 (1.9)	-0.55 (.58)
PTCI	131.7 (36.3)	135.3 (33.2)	131.0 (37.6)	-0.59 (.55)
IPSI	3.5 (1.5)	4.0 (1.2)	3.5 (1.5)	-1.77 (.08)
DERS	103.8 (27.4)	103.3 (23.9)	103.9 (28.1)	0.11 (.91)

^a^*n* = 195, ^b^*n* = 187, ^c^*n* = 188, ^d^*n* = 189, ^e^*n* = 181, ^f^*n* = 175, ^g^*n* = 167, ^h^*n* = 146, ^i^*n* = 143.

^†^High school: 12-13 years of schooling, according to the German school system; ^‡^Secondary school: 9-10 years of schooling, according to the German school system; with partner = with partner and/or child(ren) in own apartment; with parents = with parents/one parent; previous treatment = previous psychological treatment (inpatient and/or outpatient); comorbid PD = comorbid personality disorder; number of CD = number of comorbid disorders; *M, SD,* and *t* values for the clinical measures were calculated on the imputed dataset; significant effects are displayed in bold.

### Dropout in Trauma Focused-Treatment for PTSD

A total of 30 out of 195 patients (15.38%) were classified as dropouts according to our criteria.

### Analysis of Dropout Prediction

#### Association Between Dropout and Predictor Variables

Point-biserial correlations were calculated on the imputed dataset to examine the zero-order associations between dropout and the predictor variables. Results revealed a significant positive correlation between dropout and age (*r* = -.19, *p* = .02) but not between dropout and any other variable. See Supplementary Materials, Table S.1 for a complete correlation matrix of all variables studied.

#### Prediction of Dropout

To examine the unique influence of the variables of interest on dropout (0 = no dropout, 1 = dropout), a multiple logistic regression was performed on the imputed data. The results indicated that age (β = - 0.07, *p* = .04) and living situation (β = -2.16, *p* = .04) were significant predictors of dropout (see Table 2). Results showed that younger individuals were more likely to drop out of treatment, with an *OR* of 0.94. Patients who lived with their parents were at lower risk of dropout compared to those who lived alone (*OR* = 0.12).

**Table 2:** Results of the Logistic Regression Analysis

Variable	β	*SE*	*t*	*OR*	*LL*	*UL*	*p*
**Intercept**	-0.46	2.13	-0.22	0.63	0.01	47.31	.82
**Gender (Ref. = female)**	0.87	0.65	1.34	2.39	0.67	8.59	.18
**Age**	-0.07	0.03	-2.12	0.94	0.88	1.00	**.04**
Marital status (Ref. = single)							
Married	-0.33	0.67	-0.49	0.72	0.19	2.73	.63
Divorced/widowed	0.35	0.91	0.39	1.42	0.23	8.79	.70
Living situation (Ref. = alone)							
With partner	-0.11	0.68	-0.17	0.89	0.23	3.42	.87
With parents	-2.16	1.02	-2.11	0.12	0.02	0.88	**.04**
Other	0.05	0.83	0.06	1.05	0.20	5.41	.95
Highest education level (Ref. = uni. degree)							
High school	1.12	0.83	1.35	3.07	0.59	15.90	.18
Secondary school	0.45	0.79	0.57	1.57	0.33	7.43	.57
Other	0.99	1.10	0.90	2.68	0.31	23.41	.37
**Previous treatment (Ref. = no)**	-0.39	0.54	-0.73	0.68	0.23	1.97	.47
**Comorbid PD (Ref. = yes)**	0.92	0.93	0.99	2.52	0.39	16.30	.33
**Number of CD**	0.03	0.31	0.08	1.03	0.52	2.02	.93
**Gender match (Ref. = match)**	-0.20	0.62	-0.33	0.82	0.24	2.80	.74
**Approval therapist (Ref. = licensed)**	-0.02	0.51	-0.05	0.98	0.36	2.66	.96
Clinical measures							
PCL-5	-0.01	0.02	-0.34	0.99	0.95	1.04	.73
CTQ-28	-0.01	0.01	-0.81	0.99	0.96	1.02	.41
IIP-32	0.02	0.58	0.04	1.02	0.32	3.26	.97
DES	-0.04	0.19	-0.22	0.96	0.66	1.40	.83
PTCI	0.01	0.01	0.66	1.01	0.99	1.03	.51
IPSI	0.47	0.27	1.72	1.60	0.93	2.76	.09
DERS	-0.02	0.02	-1.12	0.98	0.95	1.01	.26

*Note.* Ref. = reference category; with partner = with partner and/or child(ren) in own apartment; with parents = with parents/one parent; uni. degree = university degree; previous treatment = previous psychological treatment (inpatient and/or outpatient); comorbid PD = comorbid personality disorder; number of CD = number of comorbid disorders; *OR* = Odds ratio; lower and upper CI refer to the corresponding 95% confidence intervals of the *OR*; significant effects are displayed in bold.

#### Prediction Performance

Using leave-one-out cross-validation on the imputed datasets, we evaluated the prediction performance of the logistic regression model in distinguishing between people who dropped out vs. those who did not dropout from the treatment. The model showed an accuracy of 80.5%. This accuracy score should be interpreted carefully as the data was not balanced between dropout (15.38%) and no dropout (84.62%). Indeed, the specificity was excellent (95.2%) although the sensitivity was poor (3.3%), meaning that the model is not good at identifying dropouts. ROC analysis showed an AUC value of 0.58, indicating the marginal discriminatory power of the logistic regression model.

## Discussion

The first aim of the present study was to investigate the frequency of dropout in trauma-focused, guideline-recommended interventions for PTSD in routine clinical care. 15.38% of patients unilaterally decided to prematurely terminate a started PTSD treatment. The dropout rate found in our study was considerably lower than previous estimates in routine clinical care. This applies for a sample of veterans (e.g., 36%, Goetter et al., 2015), as well as for a joint consideration of trauma-focused treatments for PTSD in RCTs and non-RCTs (e.g., 41.5%, Mitchell et al., 2022). The present findings are further accentuated by the fact that the estimated dropout rate is comparable or even slightly lower than mean dropout rates reported in meta-analyses of highly standardized RCTs, e.g., 16% for a wide range of PTSD treatments (Lewis et al., 2020) and 20.9% from guideline-recommended PTSD treatment (Varker et al., 2021). This finding on the low dropout rate is of particular importance as in clinical practice it is a major therapeutic goal to develop not only effective but also acceptable and feasible treatments. A number of possible explanations for the low dropout rate in our study are conceivable. First treatment was delivered in university-based outpatient centers which provide a well-structured treatment approach along with close supervision, while also allowing for some flexibility in treatment provision. Thus, it could be argued that the present setting combines the strengths of both, RCTs and a naturalistic setting. Note, however, that in RCTs across disorders higher dropout rates were found in university-based institutions (Swift & Greenberg, 2012). Second, therapists in training might invest more time and effort to tailor treatment to their patients’ needs than it is usually observed in regular care. Third, the manualized TF-CBT provided as a treatment may have been a particularly suitable form of treatment for the PTSD patients who participated in the current study. Conceivable explanations include the modularized phase-based approach with high flexibility in the selected modules per phase and flexibility in the sessions provided per module and phase. It is further conceivable that the specialization of the outpatient centers in PTSD treatment has an additional effect. Forth, we used well defined criteria to operationalize dropout (therapist decision combined with patient-initiated dropout).

The second aim of the study was to investigate predictors of dropout in trauma-focused, guideline-recommended interventions for PTSD in routine clinical care. A multiple logistic regression revealed age and living situation to be significant predictors, with higher risk of dropout in younger individuals and lower risk of dropout in patients who lived with their parents as opposed to living alone. The finding of younger age being predictive for dropout adds to previous findings on predictors of dropout in the general and PTSD-specific literature (Goetter et al., 2015; Swift & Greenberg, 2012), with only few studies not replicating these findings (e.g., Varker et al., 2021). Note, that all patients in the study were adults (between 18 and 65 years). Possible explanations include the fact that young patients may have more competing time demands (Goetter et al., 2015), treatment may not sufficiently match their needs, or young patients may face a lack of stability in their living environments (de Soet et al., 2024). In addition, it is conceivable that young adults have not yet experienced that PTSD symptoms in most cases do not simply disappear on their own over time (Morina et al., 2014).

To our knowledge, no previous study has investigated the influence of living situation on premature termination of treatment. Note that although patients living with their parents probably tend to be younger, the significant findings on lower risk of dropout in patients who lived with their parents compared to living alone had a unique effect, i.e., when controlling for the influence of age. To explain our findings, it appears important to address the influence of parental support on treatment outcomes. In their review of dropout in adolescents, de Soet et al. (2024) showed that parental approval, participation, and support were associated with lower risk of dropout. Therefore, young patients living with their parents might perceive more parental support and thus dropout becomes less likely than if these patients were living alone. However, more research is needed to understand the influence of living situation on premature termination of treatment.

We also examined the possible role of several clinical variables as predictors of dropout. Results showed that baseline symptom levels and associated clinical variables were overall not predictive of dropout. This is in line with earlier findings (mostly based on data collected using RCT designs) showing that e.g., symptom severity (Varker et al., 2021) or comorbidity (Mitchell et al., 2022; Snoek et al., 2021; Varker et al., 2021) were not predictive of dropout. A notable exception is a study by Mitchell et al. (2022), which did find higher PTSD symptom severity at baseline predicted dropout; however, this was only the case for clinician-rated PTSD severity and not for self-rated PTSD scores. Thus, the role of baseline PTSD symptom severity on dropout needs to be examined in further research focused on a possible role of methodological variables.

With regard to the impact of therapist characteristics on dropout our findings indicate that neither the experience level nor the gender match of the therapist has a significant influence on the dropout rate. This contradicts previous findings on treatment dropout across disorders. There is substantial evidence for the so-called therapist effect, which states that differences between therapist influence dropout rates (Deisenhofer et al., 2024; Saxon et al., 2017; Zimmermann et al., 2017). In addition, research has indicated an effect of therapist experience level on dropout (Roos & Werbart, 2013; Swift & Greenberg, 2012). For PTSD treatment in particular, evidence is sparse, with initial evidence for a therapist effect on dropout (Sayer et al., 2022). In the present study possible influences of therapist characteristics might have been minimized by the fact that patients were treated in a highly specialized service with close supervision, and the fact that most therapist were at an early-career stage. Therefore, the variability of therapist characteristics may have been rather low in the current study. In line with this reasoning, Deisenhofer et al. (2024) found that the therapist effect on dropout was significantly reduced by such institution effects.

Although it was not the primary focus of the current study, we additionally tested how well the logistic regression model would predict dropout. Taking the given imbalance between dropout and no dropout into account, the model comprising different pretreatment variables was not successful in predicting whether a patient who just started treatment would dropout during the course of treatment. Our results are in line with Vöhringer et al. (2020) who reported poor results on the discriminative power of pretreatment variables to distinguish between dropouts and completers. However, Bremer-Hoeve et al. (2023) were able to predict dropout in PTSD treatment using machine learning techniques.

In sum, only very few variables assessed in the current study were significant predictors of dropout, and the overall model could not predict dropout to a practically useful level. This is broadly in line with the majority of earlier findings. Thus, therapists and researchers should be cautious about making confident predictions about retention based on baseline data.

### Limitations

This study has a number of important strengths. One major strength is the naturalistic setting of the study, which allows for flexibility and variance in the trauma-focused, guideline-recommended treatment provided. In addition, the naturalistic setting contributes significantly to an increase in external validity and generalizability of the results to clinical practice. Nevertheless, there are a number of noteworthy limitations. First, the number of participants included in the analysis was limited, potentially leading to reduced statistical power. Even though we combined data from three outpatient centers, we had to exclude a substantial number of participants. This was due to the strict inclusion criteria regarding PTSD diagnosis and missing data for the assessment of dropout despite extensive file analysis. Second, treatments were not standardized but allowed for some flexibility based on a manual delineating key treatment principle. On the one hand, this can be regarded as a strength of the study as it is typical for routine clinical practice, where manuals are usually less strictly applied than in RCT research. On the other hand, however, we cannot rule out the possibility that the variability in the composition and timing of the use of different treatment modules may have obscured effects of certain variables in predicting dropout, as therapists may have counter-acted these variables in treatment. Third, results could be limited by the method used to operationalize dropout. Forth, the uncontrolled study design allows a more naturalistic investigation of dropout. However, in contrast to an RCT design the internal validity of effects of different variables on dropout is low. Specifically, it remains unclear whether confounding variables that were not controlled may have influenced on the occurrence of dropout. Last, although we examined a wide range of variables, potentially important aspects are missing in our dataset. These include type of trauma experienced, treatment characteristics (e.g., session frequency), and patterns of change during treatment (e.g., rate of improvement).

### Conclusion and Future Directions

In conclusion, this study provides important knowledge about the dropout rate and predictors of dropout in trauma-focused, guideline-recommended interventions for PTSD in routine clinical care. Results show that the dropout rate in this naturalistic study was comparable to dropout rates found in RCTs. In addition, two baseline predictors of dropout were identified, suggesting that young adults with PTSD may need close, supportive care, especially when they are no longer living with their parents. Therapists can act as supportive guides, build and strengthen hope (Swift & Greenberg, 2012), and be aware of urgent crises and the social needs of their young patients.

Possibly most importantly, however, our findings replicate earlier results showing that identifying patients at risk of dropping out of treatment early-on by baseline variables is challenging and currently not possible at a practically useful level. A number of implications can be drawn from this finding. First, from an applied perspective, these findings contradict widespread clinical beliefs about trauma-focused interventions being less acceptable to patients with high symptom severities, high comorbidity, or complex symptom presentations (e.g., emotion dysregulation, dissociation, interpersonal difficulties). Neither earlier research nor our current findings suggest that patients with these particularly severe and/or complex presentations are more likely to drop out of treatment. However, larger samples may provide more power and enable us to examine even a broader scope of potential predictor variables with modern machine learning approaches (see Taubitz et al., 2022). Second, the cumulated findings may suggest that it is necessary to look beyond pretreatment factors when predicting dropout and to additionally include variables investigating processes occurring in the course of treatment. For example, Zandberg et al. (2016) found that the rates of symptom change had a significant influence on dropout in patients with comorbid PTSD and alcohol dependence. Patients with low baseline symptom severity showed low risk for dropout in slow improvement and higher risk in fast improvement. When baseline symptom severity was high, the effect was u-shaped, with high risk of dropout in both slow and fast improvement (Zandberg et al., 2016). Third, further research should focus on investigating additional variables characterizing the treatment process, in particular the frequency of sessions provided. In a large-scale meta-analysis Hoppen et al. (2023) showed lower dropout rates for trauma-focused treatments delivered in high intensity. These findings are in line with Levinson et al.’s (2022) meta-analytical findings on dropout from PE provided in an outpatient setting. Finally, as earlier evidence has been inconsistent, we followed an exploratory research approach. Therefore, further studies are needed to test specific hypotheses based on theory. In addition, it appears recommendable to systematically assess subjective reasons from the patients’ perspective (Vöhringer et al., 2020).

Expanding research into dropout from PTSD treatment in these ways appears highly relevant since dropout continues to be an important clinical challenge preventing a considerable subgroup of treatment-seeking PTSD sufferers from receiving effective treatment. A better understanding of predictors of – and ultimately causal factors involved in – dropout may ultimately help to develop preventive strategies to reduce dropout and keep patients with severe symptoms in effective treatment.

## Supplementary Materials

The Supplementary Materials contain a full correlation matrix of all variables studied (see Semmlinger et al., 2025S).



SemmlingerV.
TakanoK.
WolkensteinL.
Krüger-GottschalkA.
KuckS.
DyerA.
PittigA.
AlpersG. W.
EhringT.
 (2025S). Supplementary materials to "Dropout from trauma-focused treatment for PTSD in a naturalistic setting"
[Full correlation matrix of all variables studied]. PsychOpen. 10.23668/psycharchives.15955
PMC1196057240177333

## Data Availability

The authors have no permission to share the data. The code is available upon reasonable request.

## Reporting Guidelines

We report how we determined our sample size, all data exclusions (if any), all manipulations, and all measures in the study, and we follow JARS.

## Related Versions

This publication forms part of the doctoral thesis of Verena Semmlinger at the LMU Munich.

Semmlinger, V. (2024). *The complexity of treatment failure – Prevalence and predictors of dropout and non-response in psychological treatment for traumatized populations* [Doctoral dissertation, LMU Munich]. Electronic Theses Repository of LMU Munich. https://doi.org/10.5282/edoc.34387

## Preregistration

This study’s design and its analysis were not pre-registered.

## Social Media Accounts

@thomasehring.bsky.social
